# Genomic Insights into a Colistin-Resistant Uropathogenic *Escherichia coli* Strain of O23:H4-ST641 Lineage Harboring *mcr-1.1* on a Conjugative IncHI2 Plasmid from Egypt

**DOI:** 10.3390/microorganisms9040799

**Published:** 2021-04-10

**Authors:** Azza S. Zakaria, Eva A. Edward, Nelly M. Mohamed

**Affiliations:** Microbiology and Immunology Department, Faculty of Pharmacy, Alexandria University, Alexandria 21500, Egypt; eve.farid@alexu.edu.eg (E.A.E.); nelly.mohamed@alexu.edu.eg (N.M.M.)

**Keywords:** colistin resistance, *mcr-1*, multidrug resistant uropathogenic *E. coli*, IncHI2 plasmid, whole genome sequencing, Egypt

## Abstract

The reintroduction of colistin, a last-resort antibiotic for multidrug-resistant pathogens, resulted in the global spread of plasmid-mediated mobile colistin resistance (*mcr*) genes. Our study investigated the occurrence of colistin resistance among *Escherichia coli* isolated from patients with urinary tract infections admitted to a teaching hospital in Egypt. Out of 67 isolates, three isolates were colistin-resistant, having a minimum inhibitory concentration of 4 µg/mL and possessing the *mcr-1* gene. A double mechanism of colistin resistance was detected; production of *mcr-1* along with amino acid substitution in PmrB (E123D and Y358N) and PmrA (G144S). Broth mating experiments inferred that *mcr-1* was positioned on conjugative plasmids. Whole-genome sequencing of EC13049 indicated that the isolate belonged to O23:H4-ST641 lineage and to phylogroup D. The *mcr-1*-bearing plasmid corresponded to IncHI2 type with a notable similarity to other *E. coli* plasmids previously recovered from Egypt. The unbanned use of colistin in the Egyptian agriculture sector might have created a potential reservoir for the *mcr-1* gene in food-producing animals that spread to humans. More proactive regulations must be implemented to prevent further dissemination of this resistance. This is the first characterization of *mcr-1*-carrying IncHI2:ST4 plasmid recovered from *E. coli* of a clinical source in Egypt.

## 1. Introduction

Urinary tract infections (UTIs) have been categorized amongst the most frequently encountered pathological conditions affecting 150 million people around the globe every year [1]. Although other bacteria belonging to the family *Enterobacteriaceae* can cause UTIs, uropathogenic *Escherichia coli* (UPEC) arises as the predominant etiologic agent responsible for more than 80% of UTI cases universally [2]. The emergence of a multidrug-resistant (MDR) phenotype among UPEC over recent decades worldwide is alarming and has been strongly correlated with the inappropriate empiric antimicrobial therapy [3]. Due to this escalating problem of the widespread of MDR UPEC pathogens, coupled with the exhausted antibiotic invention pipeline, colistin has been reintroduced into clinical practice after being classified by the WHO as one of the antibiotics of critical importance in human clinical settings [4]. Colistin is a polycationic peptide capable of binding to the anionic lipopolysaccharides located in the outer membrane of the Gram-negative cell wall, thus resulting in cell lysis [5]. Intrinsic colistin resistance has been linked to chromosomal mutations in the genes encoding the PmrA/PmrB and PhoP/PhoQ two-component systems or the negative regulator MgrB resulting in alterations of the lipid A molecule, the principal target of colistin [6]. In 2015, a plasmid-mediated colistin resistance gene, *mcr-1*, encoding phosphoethanolamine transferase, was identified on a conjugative IncI2 plasmid in China [7]. To date, *mcr-1* gene has been found carried on IncI2, IncX4, IncFI, IncFII, IncFIB, IncHII, IncHI2, IncP, and IncY plasmid types [8], accelerating its transmission between different bacterial species. In addition, this gene could easily disseminate from livestock, where colistin is used as a treatment or as a growth promoter in food-animal production, to humans through horizontal gene transfer [9]. The irresponsible use of colistin in veterinary practice, especially in the absence of strict legislations has contributed to the global spread of the *mcr-1* gene in 10% of animal isolates and in 0.1–2% of human isolates [5]. Despite ample information on the distribution of *mcr-1* gene worldwide, very few studies have investigated the prevalence of this gene in *E. coli* of human origin in Egypt where two *mcr-1*-postive *E. coli* strains from clinical settings have been reported so far [10,11]. In the present study, we aimed to shed light on the occurrence of colistin resistance among *E. coli* isolated from patients with UTIs in Alexandria, Egypt and to investigate the underlying mechanisms of this resistance. Additionally, we are describing the genomic features of an MDR UPEC strain belonging to ST641 clone and O23:H4 serotype, harboring the *mcr1.1* gene on an IncHI2 plasmid. This is the first complete sequence of *mcr-1*-carrying IncHI2 plasmid recovered from an *E. coli* strain with a clinical source in Egypt.

## 2. Materials and Methods

### 2.1. Bacterial Strains Collection and Identification

A total of 67 *E. coli* clinical isolates were collected through the routine laboratory facility of Alexandria Main University Hospital (AMUH) from the urine cultures of patients admitted to the hospital with UTIs over the period of June to December 2019. AMUH is a 1500-bed main referral hospital in the northern sector of Egypt with approximately 100,000 total hospital admissions per year. Samples were streaked onto MacConkey (Oxoid) and eosin methylene blue (Oxoid, Hampshire, UK) agar plates. Following incubation at 37 °C for 24 h, the isolated colonies were identified by Gram staining then subjected to standard biochemical tests including triple-sugar iron, citrate utilization and urease tests. The colistin-resistant isolates were further identified by the Vitek^®^ 2 Advanced Expert System™ (BioMérieux, La-Balme-les-Grottes, France).

### 2.2. Antimicrobial Susceptibility Testing

The susceptibility of the 67 isolates to amoxicillin-clavulanate, cefepime, cefotaxime, ceftazidime, ciprofloxacin, levofloxacin, gentamicin, imipenem, meropenem, doxycycline, sulfamethoxazole-trimethoprim and colistin was determined by the disk diffusion method. The results were interpreted according to Clinical Laboratory Standards Institute (CLSI, 2020), except for colistin, where the disk manufacturer’s guidelines (Oxoid, Hampshire, UK) were used to interpret the results [12]. The phenotype of the *E. coli* isolates was defined as MDR based on the International Expert proposal for Interim Standards Guidelines [13]. Minimum inhibitory concentration (MIC) of colistin (colistin sulfate, Sigma Chemical, St. Louis, USA) against the collected 67 *E. coli* isolates was detected by the broth microdilution in triplicates using cation-adjusted Muller-Hinton broth (Difco-BBL, Detroit, MI, USA) in accordance with the protocols recommended in M100-S30 of the 2020 CLSI which considered isolates with a colistin MIC value of ≥4 μg/mL to be resistant [12]. The *E. coli* ATCC 25922 was included as a quality control strain.

### 2.3. Detection of Antibiotic Resistance Genes by Polymerase Chain Reaction (PCR)

*E. coli* isolates with a colistin MIC value of >2 μg/mL were screened using PCR for the presence of mobile colistin resistance genes, *mcr-1* and *mcr-2* [14], and the following chromosomally encoded genes related to colistin resistance: *pmrA*, *pmrB*, *PhoP*, *PhoQ*, *mgrB* and *pmrD* [15]. Other plasmid-borne genes conferring resistance to extended-spectrum β-lactams such as *bla_SHV_* and *bla_TEM_* [16], carbapenems, *bla_NDM_* [17], and fluoroquinolones, *qnrB* [16], were analyzed as well. The sequences of the used primers obtained from Willowfort, UK, are summarized in Table 1. Genomic DNA from colistin-susceptible *E. coli* ATCC 25922 was used as the negative control. The sizes of the PCR products were determined by comparison with a molecular-sized standard (GeneRuler^TM^ 1 kb and 100 bp DNA ladder, Thermo Fisher Scientific, Hampshire, UK). Amplified DNA fragments corresponding to *pmrA*, *pmrB*, *PhoP*, *PhoQ*, *mgrB* and *pmrD* were purified using Zymo Research^TM^ DNA Purification Kit and sequenced in both directions using a system from LGC Co. Ltd. (Berlin, Germany). Mutations were determined by alignment with reference genome *E. coli* K-12 MG1655 (# NC_000913.3648.1) using NCBI BlastX tool.

### 2.4. Conjugation Experiment

Transferability of plasmid by conjugation was determined by mating Luria–Bertani (Oxoid, Hampshire, UK) broth cultures of colistin-resistant donor strains with rifampicin-resistant *E. coli* K-12 recipient at 37 °C and incubating overnight. Transconjugants were selected on MacConkey agar (Oxoid, Hampshire, UK)) supplemented with rifampicin (100 μg/mL) and colistin (4 μg/mL) [18] then tested for susceptibility to previously mentioned antimicrobials. The MIC of colistin for recipient and transconjugants was determined using the broth microdilution method as described earlier. The presence of *mcr-1* and the co-transfer of other resistance genes (*bla_SHV_*, *bla_TEM_*, *bla_NDM_* and *qnrB*) were confirmed by PCR amplification using extracted plasmids (QIAGEN Plasmid Mega Kit, Netherlands) as DNA templates and the previously mentioned primers.

### 2.5. Whole Genome Sequencing (WGS) and Bioinformatics Tools

Genomic DNA was extracted from overnight culture of *E. coli* strain EC13049 using an Invitrogen Easy-DNA^TM^ kit (Invitrogen, San Diego, CA, USA), and DNA concentration was determined using the Qubit^TM^ dsDNA BR assay kit (Invitrogen, San Diego, CA, USA). The genomic DNA was prepared for Illumina pair-end sequencing using the Illumina Nextera XT DNA Library Prep Guide Document # 15031942 v05 May 2019 following the protocol (Nextera XT DNA Library Prep Kit Reference Guide (15031942) (illumina.com)) (accessed on 18 August 2020)). The library was sequenced on an Illumina MiSeq using MiSeq Reagent Kit v2 and 500 cycles with a Standard Flow Cell. The raw data were demultiplexed using Illumina’s bcl2fastq tool (Illumina, San Diego, CA, USA), checked for quality using the FastQC tool (BaseSpace, Illumina, San Diego, CA, USA) and then quality-trimmed (Q25) and adapter-trimmed (multiplexing and sequencing adapters) using the FastQ Toolkit (BaseSpace, Illumina, CA, USA). The raw reads were assembled using the Assembler pipeline (version 1.2) available from the Center for Genomic Epidemiology (CGE) (https://cge.cbs.dtu.dk/services/Assembler/) (accessed on 21 October 2020), which is based on the Velvet algorithms for de novo short reads assembly. The assembled sequences were analyzed to confirm the species and serotype of *E. coli* strain using the CGE pipelines; K-merResistance (version 2.2) and SeroTypeFinder (version 2.0). Following confirmation, the MLST sequence type (ST), plasmid replicons, and acquired antimicrobial resistance (AMR) genes were identified using the pipelines: MLST (version 2.0), PlasmidFinder (version 2.0), and ResFinder (version 4.0) with 95% identity and 60% minimum alignment length as thresholds, available as well from CGE. The generated contigs were annotated using NCBI Prokaryotic Genome Annotation Pipeline (PGAP) (NCBI Prokaryotic Genome Annotation Process (nih.gov) (accessed on 29 January 2021). Phylogrouping was based on analysis using Clermont typing [19]. To determine the *mcr-1*-harbouring plasmid sequence, assembled contigs from *E. coli* EC13049 were mapped against *Escherichia coli* (taxid:562) using BlastN (https://blast.ncbi.nlm.nih.gov/Blast.cgi): (accessed on 4 December 2020). The presence of the *mcr-1.1* gene was identified in node 365 in the assembled contigs and the complete sequence of the generated plasmid pEGY49_MCR1.1 in *E. coli* strain EC13049 was assembled by scaffolding several nodes while any overlap regions were manually inspected. The assembled contigs were compared to the sequences of plasmids pHNSHP45-2 (GenBank accession number KU341381), pEGY1-MCR-1 (GenBank accession number CP023143) and pEGYMCR_IncHI2 (GenBank accession number MT499884) using both BlastN and SnapGene 5.2 (Insightful Science, www.snapgene.com) (accessed on 12 January 2021). SnapGene was used for the drawing and annotation of the pEGY49_MCR1.1 plasmid, while PlasmidFinder (https://cge.cbs.dtu.dk//services/PlasmidFinder/) (accessed on 12 January 2021) was used to detect the plasmid incompatibility (Inc) groups. Insertion sequence (IS) elements of the plasmid were identified using Mobile Genetic Element finder (https://cge.cbs.dtu.dk/services/MobileElementFinder/) (accessed on 20 January 2021). The circular image and comparisons between other reported similar plasmids were performed using the BLAST Ring Image Generator (BRIG) tool (http://sourceforge.net/projects/brig) (accessed on 4 February 2021). A comparative analysis of the genetic environment surrounding *mcr-1.1* gene with previously reported ones using BlastN was performed and schematic diagrams of the genetic contexts of *mcr-1.1* gene were drawn using the SnapGene tool (GSL Biotech LLC, San Diego, CA, USA).

## 3. Results and Discussion

### 3.1. Antimicrobial Resistance Profiles and Colistin MIC

**Figure 1:**
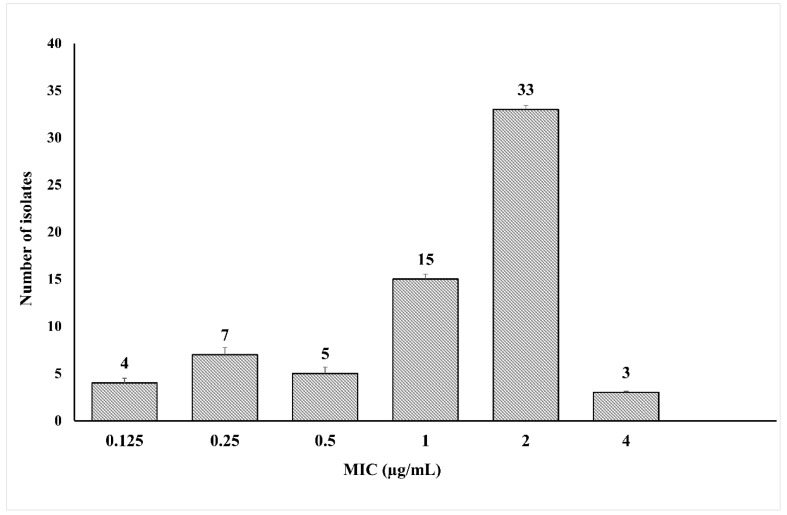
Distribution of colistin MIC values among 67 *E. coli* isolates from urinary tract infections.

The susceptibility of 67 *E. coli* strains isolated from patients with UTIs to different antibiotics showed that 89.6% of these isolates possessed an MDR phenotype being resistant to ≥3 groups of antimicrobials. All the isolates were resistant to amoxicillin-clavulanate, third and fourth generation cephalosporins. High resistance rates, exceeding 50%, were detected for fluoroquinolones, sulfamethoxazole-trimethoprim and gentamicin. While 67.2% of the isolates were resistant to imipenem, meropenem retained its activity against most of the isolates with a percentage of susceptibility of 86.6%. Meropenem is listed as a precious antibiotic in Egyptian hospital settings and is not widely prescribed in out-patient clinics. The most frequently encountered MDR profile included resistance to amoxicillin-clavulanate, cephalosporins, fluoroquinolones and sulfamethoxazole-trimethoprim (Supplementary Table S1), a profile that correlates with the extensive usage of these antibiotics in Egyptian healthcare establishments as per the Infectious Diseases Society of America (IDSA) guidelines implemented in these facilities. The prevalence of the MDR phenotype among UPEC isolates has been recently reported in different governorates in Egypt. Gawad et al. [2] detected the MDR phenotype among 91% of UPEC isolates in two governorates in the northeastern part of Egypt. In the capital, Cairo, Abdelkhalik et al. [20] reported an MDR pattern in 80% of UPEC isolated from women with acute uncomplicated cystitis. In the present study, six isolates (8.9%) were resistant to colistin. Reports from the Upper Egypt sector revealed a colistin resistance of 32.7%, 20.8% and 23.1% among UPEC isolates in three different governorates [11,21]. After a preliminary screening using disk diffusion method, the MIC of colistin against the 67 tested isolates in the current study was determined by broth microdilution, the recommended method by CLSI 2020. The colistin MIC values ranged from 0.125 to 4 µg/mL with MIC_50_ and MIC_90_ reaching 2 µg/mL (Figure 1). Using the broth microdilution method, three isolates (4.5%), EC13049, EC14142 and EC13655, had MIC values = 4 µg/mL and were selected for further tests.

### 3.2. Molecular Identification of Resistance Genes

**Figure 2:**
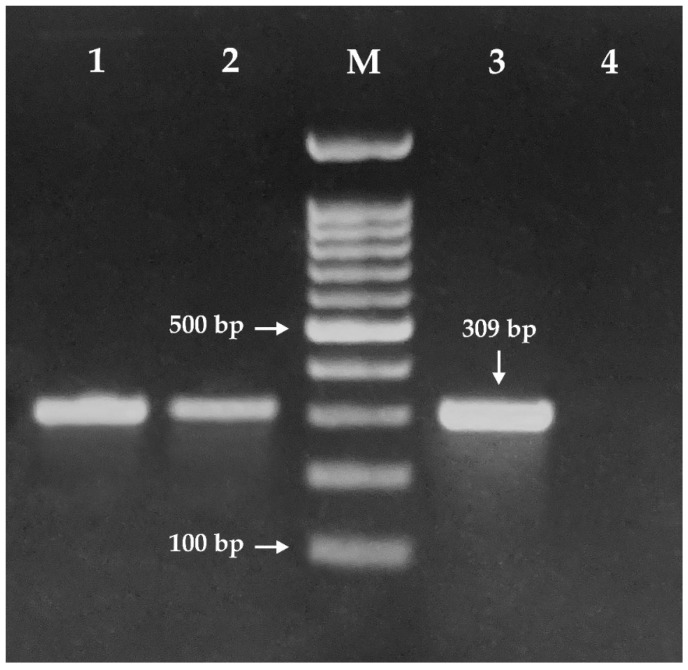
Agarose gel showing PCR amplification of *mcr-1* gene in three *E. coli* strains isolated from urinary tract infections. Lane M: DNA molecular weight marker (100 bp ladder). Lanes 1, 2 and 3 show the amplicon (309 bp) of *mcr-1* gene corresponding to EC13049, EC14142 and EC13655, respectively. Lane 4 exhibits the negative control (*E. coli*, ATCC 25922).

PCR analysis identified plasmid-mediated colistin resistance associated with the presence of the *mcr-1* gene in three MDR isolates, EC13049, EC14142 and EC13655 (Figure 2). None of the isolates harbored the *mcr-2* gene. Two *mcr-1*-positive clinical *E. coli* isolates have been identified in Egypt to date; the first one was isolated from a patient with bacteremia while the second was recovered from a cancer patient [10,11]. Nevertheless, reports on *mcr-1*-producing *E. coli* isolates recovered from livestock or food in Egypt were numerous and included those from a diseased cow [22], healthy broilers [23], cheese [24] and beef sausage [25]. The use of colistin in the Egyptian farming industry as a therapeutic and prophylactic agent is accumulating *mcr-1* in food-producing animals with a potential risk of its transfer to human food chain [26]. This imposes a challenge in a country struggling with high burden of infectious diseases and low restrictions on antibiotics access as Egypt [22]. The problem of antimicrobial resistance is aggravated when isolates presenting the *mcr-1* gene are co-expressing other genes of resistance, such as those encoding extended-spectrum β-lactamases or carbapenemases [27]. The three *mcr-1*-positive isolates in the present study co-harbored genes conferring resistance to extended-spectrum β-lactams, *bla_SHV_* and *bla_TEM_*. Isolate EC13655 possessed the carbapenemase gene *bla_NDM_*, whereas no isolates carrying the *qnrB* gene were detected (Table 2).

### 3.3. Detection of Amino Acids Alterations in Two-Component Systems; PmrAB and PhoPQ and Their Regulators, MgrB and PmrD

Acquired resistance to colistin is exerted through chromosomal mechanisms modifying the lipid A moiety of Gram-negative bacteria leading to its inactivation and consequent loss of the colistin target. These modifications are associated with mutations inthe two-component systems PhoP/PhoQ and PmrA/PmrB [28]. Mutations in *mgrB* and *pmrD* genes are also known to play a role in colistin resistance in the *Enterobacteriaceae*, where the inactivation of MgrB, the negative regulator of the PhoPQ system, leads to overexpression of the *phoPQ* operon, whereas the activation of PmrD by PhoP upregulates PmrAB [15]. To address this part, the full nucleotide sequences of *PhoP*, *PhoQ*, *pmrA*, *pmrB, mgrB* and *pmrD* from the three colistin-resistant isolates were inspected and compared to those of *E. coli* K-12 MG1655 (Table 3).

The tested isolates exhibited a wild type *mgrB*. Sequence analysis of the PhoP revealed two missense mutations (I44L and V108M). These variations are frequently reported in *E. coli* as per the available sequences in GenBank, but their impact on colistin resistance is uncomprehended [28]. Amino acid substitutions were found in two sites of PhoQ: A166V and S138T. These two mutations have not been related so far to colistin resistance [28]. The PmrA sequence revealed three amino acid substitutions, among which G144S substitution in isolate EC13655 is reported to contribute to colistin resistance [29]. Six amino acid alterations were detected in PmrB, among which E123D and Y358N mutations, located in the histidine kinase and phosphate-related domain, might affect the phosphate transfer between the two-component systems resulting in colistin-resistant isolates [29]. PmrD, which promotes the connection of PhoPQ and PmrAB two-component systems, displayed mutations at four sites (L19I, S71C, V83A and D14N), but their associations with colistin resistance have not been reported [15]. Although the double mechanism of colistin resistance, the production of *mcr-1* gene along with amino acid substitution in PmrB (E123D and Y358N) or PmrA (G144S), depicted in this study in isolates EC14142 and EC13655 is considered a rare finding, it has been reported earlier by other investigators [30].

### 3.4. Conjugative Transfer of mcr-1 Gene

Broth mating experiments and subsequent PCR analysis were performed for the *mcr-1*-positive isolates: EC13049, EC14142 and EC13655. Transconjugants were recovered from the three donors, inferring that *mcr-1* was positioned on self-transferable conjugative plasmids (Table 2). Conjugation frequencies were in the range of 10^−4^ to 10^−7^ CFU per donor cell and the values of colistin MIC for the transconjugants increased from 64- to 256-fold as compared to the recipient. The resistance phenotype and genotype of donors and transconjugants were almost identical except for the failure of transfer of gentamicin and sulfamethoxazole-trimethoprim in EC14142 and doxycycline in isolate EC13655. This suggests that the resistance determinants for these antibiotics might be located on non-transferable plasmids. A previous exposure to tetracycline seems to be required to enhance the conjugal transfer of tetracycline resistance determinants as reported earlier [31]. The conjugative transfer of the *mcr-1* gene and the co-transfer of *bla*_SHV_, *bla*_TEM_ and *bla*_NDM_ confirmed by PCR illustrate the potential risk of horizontal gene transfer to other Gram-negative bacteria, raising challenges in tailoring an adequate clinical therapy [32].

### 3.5. Genome Analysis of UPEC Strain EC13049

Since the three *mcr-1-*positive *E. coli* isolates displayed a near-identical antimicrobial resistance phenotype, the EC13049 strain, with the highest conjugation frequency, was chosen for WGS analysis by the Illumina platform. The de novo assembly revealed that the complete genome of *E. coli* EC13049 comprises a chromosome of 5,553,206bp distributed in 300 contigs with a depth coverage of 198× and an overall G + C content of 52.4% and an N50 of 69479. The assembly statistics generated through WGS are described in Supplementary Table S2. Genotyping of EC13049 indicated that the isolate belongs to the serogroup O23:H4 and to the serotype ST641 according to the MLST (Pasteur) allelic profile, which uses the sequences of the eight house-keeping genes (*dinB*, *icdA*, *pabB*, *polB*, *putP*, *trpA*, *trpB*, *uidA*) with assigned allele numbers (2, 237, 23, 15, 10, 15, 10, 12). ST641, a worldwide clone previously shown to harbor *mcr,* was reported in different geographical regions including China, Germany, and Columbia [33,34,35]. Using the sequence data, two virulence determinant-encoding genes, *vat* and *chuA*, known to enable an *E. coli* strain to colonize the urinary bladder producing UTIs, were located on the genome of EC13049, categorizing this isolate as a uropathogenic one [36]. In addition, Clermont phylotyping assigned the isolate to phylogroup D, which is typically affiliated with UTIs [19]. Typing of the *fimH* (type 1 fimbrial adhesin) gene showed that the isolate carries *fimH63* allele, resulting in the clonotype CH45-63. The resistome of the EC13049 displayed an MDR genotype carrying genes responsible for resistance to aminoglycosides (*aac(3)-IIa*, *aadA1*, *aadA2*, *aadA24*, *aadA22*, *aph(6)-Id*, *aph(3′)-Ia* and *aph(3′’)-Ib*), streptothricin (*sat1*), amphenicols (*floR*, *catA1*, *cmlA1*), sulphonamides (*sul2*, *sul3*), extended-spectrum β-lactams (*bla*_SHV-12_, *bla*_TEM-1B_), fluoroquinolones (*qnrS1*), colistin (*mcr-1.1*), tetracycline (*tetA*), lincosamide (*InuF*) and trimethoprim (*dfrA1*). A multidrug efflux pump gene (*mdfA*) mediating resistance to aminoglycosides, phenicols, fluoroquinolones, tetracycline, rifamycin and macrolides was detected as well. The expression of MdfA is stated to confer additional resistance to clinically important, chemically unrelated antibiotics even in the absence of other specific genes [30]. Furthermore, a double-serine mutation (*gyrA* S83L and *parC* S80R) and an additional substitution in *gyrA* (D87N) associated with resistance to fluoroquinolones were identified by ResFinder. Double-serine substitutions in *gyrA* and *parC* have been outlined as a dominant feature of MDR *E. coli* linked to high resistance levels to fluoroquinolones [37]. PlasmidFinder identified nine plasmid replicon types: Col(MG828), Col(pHAD28), IncB/O/K/Z, IncFIB, IncFIC (FII), IncHI2 (carrying the *mcr-1.1* gene), IncHI2A, IncQ1 and IncX1 (Table 4).

### 3.6. Genomic Location of mcr-1.1 Gene and Similarity to Published Plasmids

**Figure 3:**
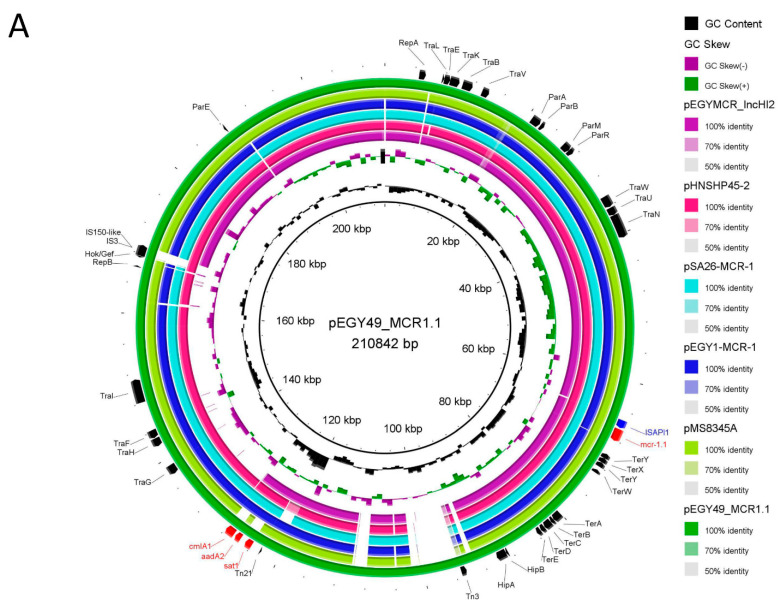

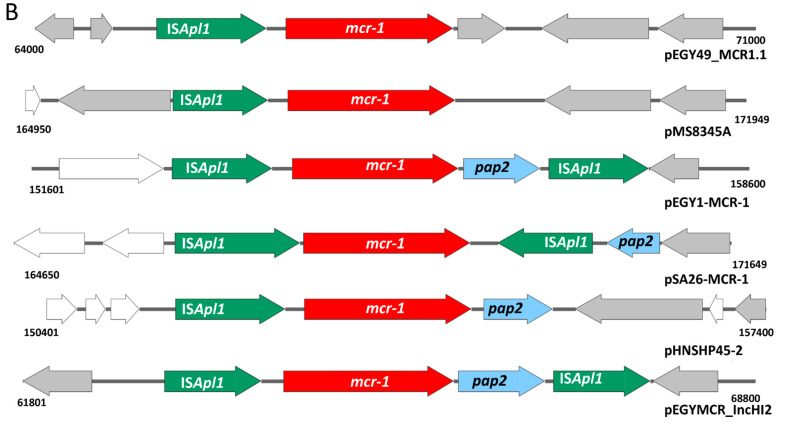
(**A**) BLAST Ring Image Generator (BRIG) visualization comparing IncHI2-*mcr-1*-positive *E. coli* plasmids. The outermost ring (dark green) corresponds to pEGY49_MCR1.1 with its size indicated in the middle of the ring. Next, pMS8345A is shown (light green). The third ring corresponds to pEGY1-MCR-1 (navy blue), then pSA26-MCR1 (aqua) and pHNSHP45-2 (pink). The innermost ring (purple) refers to pEGYMCR_IncHI2. The labels in the outer ring represent the annotation of the genes associated to virulence, antibiotic resistance, stress resistance and replicons. The *mcr-1* gene and IS*Apl1* are marked in red and blue, respectively. (**B**) Genetic environment of *mcr-1* gene in pEGY49_MCR1.1 compared to those in other IncHI2 plasmids from *E. coli*. The arrows indicate open reading frames (*orf*), with red, green, blue, grey and white arrows representing *mcr-1*, IS*Apl1* element, *pap2*, ORFs with known function, and ORFs with unknown function, respectively.

The *mcr-1.1* gene was located on an IncHI2 plasmid which was 210,842 bp in length and had an average G + C content of 45.3%. This is consistent with previous findings indicating that the global spread of *mcr-1* is mainly generated by three plasmid replicon types: IncI2, IncX4, and IncHI2 [8]. The plasmid designated pEGY49_MCR1.1 (accession number: MW719568) was shown to be a conjugative plasmid transferring the *mcr-1.1* gene to the recipient *E. coli* K-12 and was subtyped as pST4. The pEGY49_MCR1.1 plasmid contained 236 CDSs (128 hypothetical proteins) and showed the conserved IncHI2 architecture comprising genes encoding proteins involved in conjugative transfer, replication, plasmid stabilization and maintenance. Furthermore, genes corresponding to Tn*3* transposon and to the insertion sequence IS*ApI1* were located on the plasmid. pEGY49_MCR1.1 carried the tellurium resistance genes (*terABCDE*) usually associated with IncHI2 plasmids, in addition to *terX*, *terY* and *terW* [38]. Transposon Tn21 known to encode genes for heavy-metal resistance, namely mercuric compounds, was found as well on the plasmid [8]. BLASTn analysis of pEGY49_MCR1.1 with the available database showed that this plasmid shared high sequence identity (99.9% nucleotide identity, 93% sequence length) with the first reported IncHI2 *mcr-1*-positive plasmid pHNSHP45-2 (GenBank ID KU341381) identified in China [39]. In comparison with the two IncHI2 sequenced plasmids from Egypt, pEGY49_MCR1.1 showed 100% nucleotide identity (93% sequence length) to the IncHI2 pEGY1-MCR-1 (GenBank ID CP023143) plasmid identified in an *E. coli* strain isolated from a popular Egyptian raw milk cheese, karish cheese [24] and shared high similarity (99.9% nucleotide identity, 75% sequence length) to the *mcr-1*-positive IncHI2 plasmid pEGYMCR_IncHI2 (GenBank ID MT499884) identified in *E. coli* strain recovered from a chicken carcass in the Upper Egypt Sector [8]. The remarkable similarity of the *mcr-1*-carrying plasmid in *E. coli* identified from human sources in the current study to those recovered from different food origins in Egypt reflects the local epidemic nature of this IncHI2 plasmid that circulates among different sources and facilitates the dissemination of the *mcr-1* gene from the food chain to humans. pEGY49_MCR1.1 shared a striking sequence similarity to two IncHI2 *mcr-1*-bearing plasmids from clinical source identified in the Middle East region, where it was totally identical to pMS8345A (100% nucleotide identity, 92% sequence length) recovered from *E. coli* isolated from a patient in Qatar (GenBank ID CP025402) [40], and was highly similar (99.9% nucleotide identity, 92% sequence length) to pSA26-MCR1 (GenBank ID KU743384) isolated from *E. coli* strain from a patient in Saudi Arabia [41]. This similarity points to the possibility of this plasmid being the key vehicle for the spread of the *mcr-1* gene among *E. coli* from the Middle East region to North Africa, where Egypt is located. Additional genes were detected alongside *mcr-1* and were associated with resistance to aminoglycosides (*aadA2*), streptothricin (*sat1*) and amphenicols (*cmlA1*) (Figure 3A). The co-existence of other antibiotic resistance genes on *mcr-1*-carrying plasmids presents the hazard of screening colistin-resistant strains under the imposed selective pressure exerted by antibiotics other than colistin [42].

### 3.7. Genetic Environment of mcr-1.1 Gene

It has been hypothesized that in around 2006, an initial mobilization event of *mcr-1* occurred via the development of the IS*Apl1*-*mcr-1-orf-*IS*Apl1* composite transposon [43]. Following this event, the stabilization of *mcr-1* into a wide range of plasmid types was reached by the loss of flanking IS*Apl1* elements over time and the subsequent *mcr-1* spread through plasmid transfer to different bacterial species started. Although the mobilizing capability of the transposon is thought to be suppressed after the loss of both IS*Apl1* elements, the presence of a single copy is stated to be competent in partial translocation ability, with the upstream copy being more functionally efficient [44]. In silico analysis of the immediate proximity of *mcr-1.1* in the detected IncHI2 plasmid confirmed that a single upstream copy of the IS*Apl1* insertion sequence was detected in pEGY49_MCR1.1 (Figure 3B). The absence of the complete composite transposon and the presence of the upstream copy of IS*Apl1* element suggest that the *mcr-1.1* gene was transposed into the IncHI2 plasmid not long before isolation [32]. The flanking sequences of *mcr-1* in pEGY49_MCR1.1 were highly similar (99.9% nucleotide identity, 81% query coverage) to those in plasmid pMS8345A (IncHI2; CP025402), both containing a single upstream copy of IS*Apl1* element in the direct vicinity of *mcr-1*. The genetic structures surrounding *mcr-1* gene in pEGY1-MCR-1 (IncHI2; CP023143) and pEGYMCR_IncHI2 (IncHI2; MT499884) showed the presence of IS*Apl1*-*mcr-1-orf-*IS*Apl1* differing from the genetic context of *mcr-1* gene in the plasmid reported in this study, the latter lacking this composite transposon. In addition to the upstream copy of IS*Apl1* element in the flanking sequence of *mcr-1* in pHNSHP45-2 (IncHI2; KU341381), a *pap2-*like phosphatase was found directly downstream of *mcr-1* while pSA26-MCR1 (IncHI2; KU743384) exhibited a IS*Apl1-pap2-*like phosphatase insertion downstream of *mcr-1* gene (Figure 3B). Noteworthily, the comparison of the previously mentioned six genetic environments using BLASTn resulted in 99.9% identity with different query coverage.

## 4. Conclusions

In conclusion, we provide here the first report of the occurrence of an *mcr-1*-mediated colistin resistance carried on IncHI2 plasmid in an MDR UPEC of O23:H4-ST641 lineage isolated from a patient admitted to a tertiary hospital in Alexandria, Egypt. The notable similarity of this plasmid to the two IncHI2 plasmids from *E. coli* strains recovered from animal origins described in Egypt so far strongly suggests that this plasmid type is trafficking between food of animal origin and clinical environments disseminating colistin resistance. The unbanned use of colistin as an in-feed antibiotic and growth promoter in Egypt might have created a potential reservoir for the *mcr-1* gene in food-producing animals that had spread to humans. More proactive regulations to the use of this last-resort drug in the agriculture sector must be implemented to guarantee the success of the treatment and to prevent further dissemination of this resistance.

## Figures and Tables

**Table 1:** List of primer pairs used for PCR amplification of the selected genes in this study.

Target Gene	Nucleotide Sequence (5′→3′)	Amplicon Size (bp)	Reference
*mcr-1*	F: 5′-CGGTCAGTCCGTTTGTTC-3′ R: 5′-CTTGGTCGGTCTGTAGGG-3	309	[14]
*mcr-2*	F: 5′-TGTTGCTTGTGCCGATTGGA-3′ R: 5′-AGATGGTATTGTTGGTTGCTG-3′	567	[14]
*mgrB*	F: 5′-TCGTAGCGGCAATATGCGC-3′ R: 5′-GACGCAATGTTCATCACGCC-3′	385	[15]
*PhoP*	F: 5′-ATGCGCGTACTGGTTGTTG-3′ R: 5′-AGTACTACCGCTGCCGTTGCC-3′	747	[15]
*PhoQ*	F: 5′-CAGGGCTATCTGTTCGAATTGCG-3′ R: 5′-ACGGATGCTTAACGTAATGCGTG-3′	1523	[15]
*pmrA*	F: 5′-CTGATTGTTGAAGACGATACGC-3′ R: 5′-AGTTTTCCTCATTCGCGACCA-3′	657	[15]
*pmrB*	F: 5′-GAATCTGATGCATTTGCGC-3′ R: 5′-TTATATCTGGTTTGCCACGTACTG-3′	1098	[15]
*pmrD*	F: 5′-CGGTGCGCTGGCTAACTCTGCC-3′ R: 5′-AGTAGCCTGTATTATGAGCGGG-3′	527	[15]
*bla_SHV_*	F: 5′-ATT TGT CGC TTCTTT ACT CGC -3′ R: 5′-TTT ATG GCG TTACCT TTG ACC-3′	1018	[16]
*bla_TEM_*	F: 5′-CATTTCCGTGTCGCCCTTATTC-3′ R: 5′-CGTTCATCCATAGTTGCCTGAC-3′	800	[16]
*qnrB*	F: 5′-CCT GAGCGGCACTGAATTTAT-3′ R: 5′-GTT TGCTGCTCGCCAGTCGA -3′	120	[16]
*bla_NDM_*	F: 5′-GGTTTGGCGATCTGGTTTTC-3′ R: 5′-CGGAATGGCTCATCACGATC-3′	621	[17]

**Table 2:** Antimicrobial resistance profile of colistin-resistant *E. coli* clinical isolates and their transconjugants.

*E. coli* Isolates	Colistin MIC (μg/mL)	Conjugation Frequency ^a^ (CFU/Donor Cell)	Resistance Genes	Resistance Profile
EC13049	4		*mcr-1*, *bla_SHV_*, *bla_TEM_*	AMC, CTX, CAZ, FEP, SXT, CIP, LEV, CN, DO, CT
Transconjugant of EC13049	32	2.8 × 10^−4^	*mcr-1*, *bla_SHV_*, *bla_TEM_*	AMC, CTX, CAZ, FEP, SXT, CIP, LEV, CN, DO, CT, RD
EC14142	4		*mcr-1*, *bla_SHV_*, *bla_TEM_*	AMC, CTX, CAZ, FEP, IPM, SXT, CIP, LEV, CN, CT
Transconjugant of EC14142	8	5.8 × 10^−5^	*mcr-1*, *bla_SHV_*, *bla_TEM_*	AMC, CTX, CAZ, FEP, IPM, CIP, LEV, CT, RD
EC13655	4		*mcr-1*, *bla_SHV_*, *bla_TEM_*, *bla_NDM_*	AMC, CTX, CAZ, FEP, IPM, SXT, CIP, LEV, CN, DO, CT
Transconjugant of EC13655	16	1.7 × 10^−7^	*mcr-1*, *bla_SHV_*, *bla_TEM_*, *bla_NDM_*	AMC, CTX, CAZ, FEP, IPM, SXT, CIP, LEV, CN, CT, RD

^a^ The recipient was rifampicin-resistant *E. coli* K-12 with a colistin MIC of 0.125 μg/mL. AMC: amoxicillin-clavulanate; CTX: cefotaxime; CAZ: ceftazidime; FEP: cefepime; IPM: imipenem; SXT: trimethoprim-sulfamethoxazole; CIP: ciprofloxacin; LEV: levofloxacin; CN: gentamicin; DO: doxycycline; CT: colistin, RD: rifampicin.

**Table 3:** Mutations in chromosomally encoded genes related to colistin resistance in tested *E. coli* clinical isolates.

*E. coli* Isolate	Gene/Protein					
	pmrA/PmrA	pmrB/PmrB	pmrD/PmrD	PhoP/PhoP	PhoQ/PhoQ	mgrB/MgrB
EC13049	S29G ^b^	H2R ^b^, D283G ^b^, A360V ^b^	L19I ^b^, S71C ^b^, V83A ^b^	None	None	None
EC14142	S29G ^b^, T31S ^b^	H2R ^b^, E123D ^a^, D283G ^b^, V351I ^b^	D14N ^b^, S71C ^b^, V83A^b^	V108M ^b^, I44L ^b^	A166V ^b^, S138T^b^	None
EC13655	S29G ^b^, G144S ^a^	D283G ^b^, Y358N ^a^	None	V108M ^b^, I44L ^b^	S138T ^b^	None

^a^ Polymorphism reported to be associated with colistin resistance. ^b^ Reported polymorphism but its association with colistin resistance is unknown.

**Table 4:** Features, molecular typing, resistance profile and plasmid replicon types carried in UPEC EC13049 isolate from Egypt.

Serotype ^a^	Phylogroup ^b^	Pathotype ^c^	Sequence Type (ST) ^d^	Clonotype ^e^	Resistance Profile ^f^		Plasmid Replicon Type ^g^
					Antimicrobial Class	ARGs (in Black) and Point Mutations (in Blue)	
**O23:H4**	Group D	UPEC	641	CH45-63	β-lactams	*bla*_SHV-12_, *bla*_TEM-1B_	Col(MG828)
					Aminoglycosides	*aac(3)-IIa*, *aph(3′)-Ia*, *aadA1*, *aadA2*, *aadA24*, *aadA22*, *aph(6)-Id*, *aph(3″)-Ib*, *mdfA*	Col(pHAD28)
					Streptothricin	*sat1*	IncB/O/K/Z
					Phenicol	*floR*, *catA1*, *cmlA1*, *mdfA*	IncFIB
					Fluoroquinolones	*qnrS1, mdfA*, *gyrA* S83L, *gyrA* D87N, *parC* S80R	IncFIC (FII)
					Colistin	*mcr-1.1*	**IncHI2**
					Folate pathway antagonist	*sul2*, *sul3, dfrA1*	IncHI2A
					Tetracycline	*tetA*, *mdfA*	IncQ1
					Lincosamide	*InuF*	IncX1
					Rifamycin	*mdfA*	
					Macrolide	*mdfA*	

^a^ Data obtained from SerotypeFinder version 2.0. ^b^ Based on ClermonTyping method. ^c^ Pathotype: as determined by the presence of *chuA* and *vat* virulence genes. ^d^ ST: sequence type, data obtained from MLST version 2.0. ^e^ Clonotype: determined according to *fumC*- *fimH* alleles, data obtained from CHTyper version 1.0. ^f^ ARGs: Antimicrobial resistance genes and point mutations as obtained from ResFinder version 4.0. ^g^ Data represent plasmid incompatibility (Inc) group designations as determined by PlasmidFinder version 2.0. Bold formatting indicates the plasmid carrying the *mcr-1.1* gene.

## Data Availability

The EC13049 whole-genome shotgun sequence was deposited in DDBJ/ENA/GenBank under the BioProject accession number JAENHQ000000000 (https://submit.ncbi.nlm.nih.gov/subs/wgs/JAENHQ000000000 (accessed on 9 April 2021)). The raw sequence data have been submitted to the Sequence Read Archive (SRA)Top of Form (https://submit.ncbi.nlm.nih.gov/about/sra/ (accessed on 9 April 2021)) under study accession number PRJNA688626 (https://submit.ncbi.nlm.nih.gov/subs/sra/ PRJNA688626). The plasmid data were deposited in NCBI using Banklt tool under the accession number MW719568.

## Supplementary Materials

The following are available online at https://www.mdpi.com/article/10.3390/microorganisms9040799/s1, Table S1: Antimicrobial resistance profile for 67 *E. coli* strains isolated from urinary tract infections where blue boxes represent resistance and white boxes indicate susceptibility to the corresponding antimicrobial disk. Table S2: Assembly statistics generated through WGS of *E. coli* strain EC 13049.
