# Supplementary material for: Genomic Insights into a Colistin-Resistant Uropathogenic Escherichia coli Strain of O23:H4-ST641 Lineage Harboring mcr-1.1 on a Conjugative IncHI2 Plasmid from Egypt
Source: Microorganisms. 2021 Apr 10;9(4):799. doi: 10.3390/microorganisms9040799 (PMC8069611; doi:10.3390/microorganisms9040799)
Supplement: Supplementary file 1 [file microorganisms-09-00799-s001.pdf]

Supplementary Table S1: Antimicrobial resistance profile for 67 *E. coli* strains isolated from urinary tract infections where blue boxes represent resistance and white boxes indicate susceptibility to the corresponding antimicrobial disk.

Isolate Code	AMC 30	CTX 30	CAZ 30	FEP 30	CIP 5	LEV 5	SXT 25	CN 10	DO 30	IPM 10	MEM 10	CT 10
EC11377	■	■	■	■	■	■		■		■		
EC11998	■	■	■	■	■	■	■	■	■	■		
EC12665	■	■	■	■	■	■	■	■		■		
EC11994	■	■	■	■	■	■	■		■			
EC11856	■	■	■	■	■	■			■	■		
EC11804	■	■	■	■	■	■						
EC12220	■	■	■	■	■	■	■	■	■	■		
EC11443	■	■	■	■	■	■	■	■	■	■	■	■
EC11322	■	■	■	■	■	■	■		■	■		
EC11442	■	■	■	■	■	■		■				
EC11376	■	■	■	■	■	■				■		
EC12181	■	■	■	■	■	■	■			■		
EC11997	■	■	■	■	■	■	■	■	■	■		■
EC11384	■	■	■	■	■	■	■			■		
EC11302	■	■	■	■	■	■		■		■		
EC2031	■	■	■	■	■	■						
EC11868	■	■	■	■	■	■		■	■	■		
EC13070	■	■	■	■	■	■	■		■	■		
EC12911	■	■	■	■	■	■	■		■	■		
EC12946	■	■	■	■	■	■	■		■	■	■	
EC12947	■	■	■	■	■	■	■		■	■		
EC13049	■	■	■	■	■	■	■	■	■			■
EC13205	■	■	■	■	■	■	■	■	■	■		
EC12825	■	■	■	■	■	■	■	■	■	■	■	
EC12758	■	■	■	■	■	■		■		■		
EC12665	■	■	■	■	■	■	■	■	■	■		
EC15635	■	■	■	■								
EC12693	■	■	■	■	■	■	■	■	■	■		
EC15984	■	■	■	■	■	■	■	■		■		
EC16073	■	■	■	■	■	■	■			■		
EC12638	■	■	■	■						■		
EC12572	■	■	■	■	■	■		■		■		
EC13213	■	■	■	■						■		
EC12330	■	■	■	■	■	■	■					
EC12319	■	■	■	■						■		
EC14051	■	■	■	■	■	■	■	■	■	■		
EC16585	■	■	■	■	■	■	■	■		■	■	■
EC13495	■	■	■	■	■	■	■	■	■	■	■	
EC14657	■	■	■	■	■	■	■	■	■	■		
EC13933	■	■	■	■	■	■	■	■	■	■		
EC13791	■	■	■	■	■	■	■	■	■	■		
EC14437	■	■	■	■	■	■	■					
EC16726	■	■	■	■	■	■	■	■		■		
EC13728	■	■	■	■	■	■	■		■			

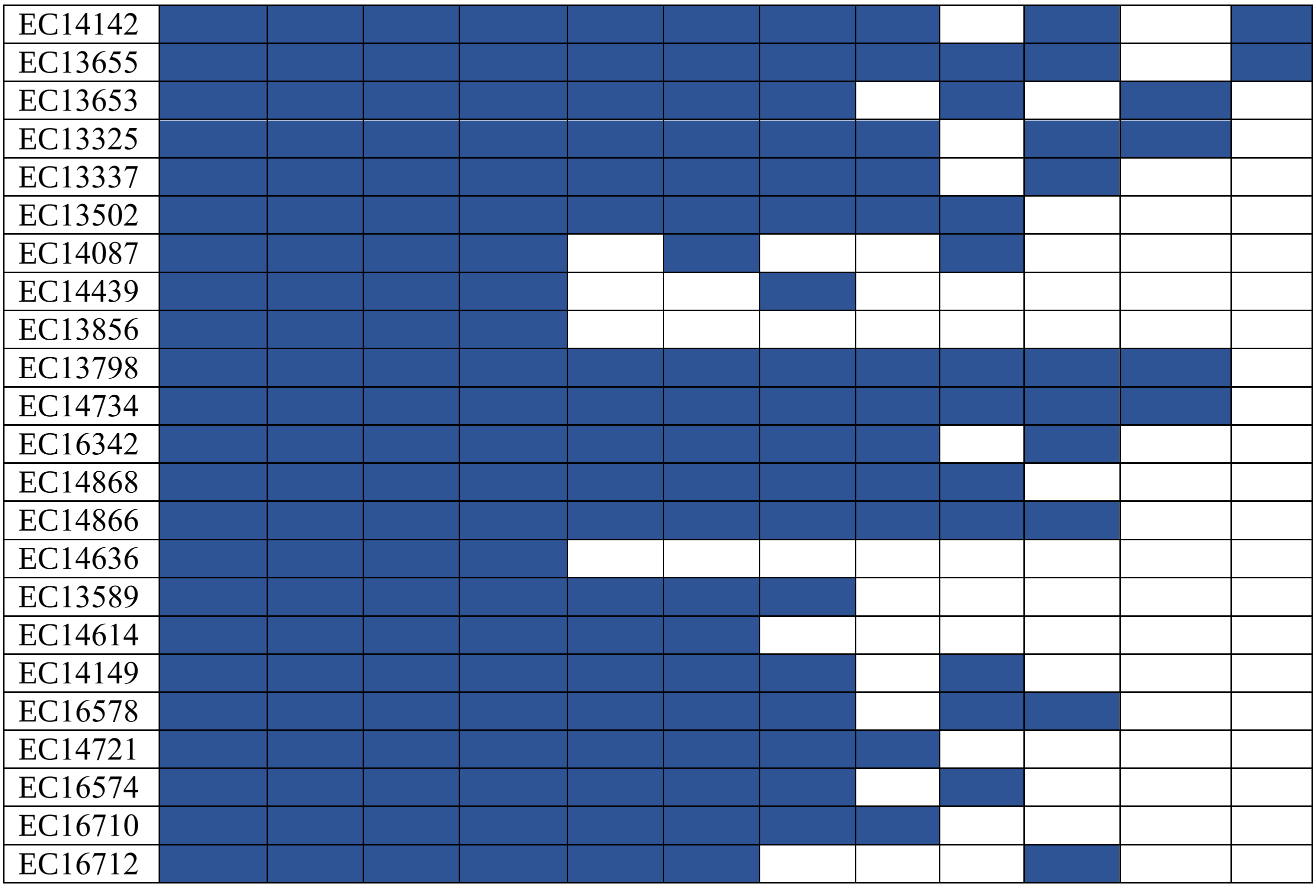

AMC: amoxicillin-clavulanate; CTX: cefotaxime; CAZ: ceftazidime; FEP: cefepime; CIP, ciprofloxacin; LEV: levofloxacin; SXT: trimethoprim-sulfamethoxazole; CN: gentamicin; DO: doxycycline; IPM: imipenem; MEM: meropenem; CT: colistin.

Supplementary Table S2: Assembly statistics generated through WGS of *E. coli* strain EC13049.

Depth of coverage	198x
Genome breadth (%)	99.8
Number of reads	4,947,285
Total length of sequences	5,553,206
Total number of contigs	300
N50 (bp)	69479
GC (%)	52.4
CDSs	4,883
5s, 16s, 23rrna	1, 1, 1
